# Chromosome-level genome assembly of the giant ladybug *Megalocaria dilatata*

**DOI:** 10.1038/s41597-024-02990-1

**Published:** 2024-01-24

**Authors:** De-Qiang Pu, Xing-Long Wu, Zhi-Teng Chen, Shu-Jun Wei, Peng Cai, Hong-Ling Liu

**Affiliations:** 1 Institute of Plant Protection, Sichuan Academy of Agricultural Sciences, Chengdu, 610066 China; 2 School of Grain Science and Technology, Jiangsu University of Science and Technology, Zhenjiang, 212004 China; 3 Institute of Plant and Environmental Protection, Beijing Academy of Agriculture and Forestry Sciences, Beijing, 100097 China; 4 Horticultural Institute, Sichuan Academy of Agricultural Sciences, Vegetable Germplasm Innovation and Variety Improvement Key Laboratory of Sichuan Province, Chengdu, 610066 China

**Keywords:** Evolutionary genetics, Transcriptomics, Comparative genomics, Genome

## Abstract

The giant ladybug *Megalocaria dilatata* (Fabricius) is a potential biocontrol agent and a valuable model for coccinellid genomics and evolutionary biology. However, the lack of a reference genome for *M. dilatata* has impeded further explorations into its evolution and constrained its use in pest management. Here, we assembled and annotated a high-quality, chromosome-level genome of *M. dilatata*. The resulting assembly spans 772.3 Mb, with a scaffold N50 of 72.48 Mb and a GC content of 34.23%. The Hi-C data aided in anchoring the assembly onto 10 chromosomes ranging from 43.35 to 108.16 Mb. We identified 493.33 Mb of repeat sequences, accounting for 63.88% of the assembled genome. Our gene prediction identified 25,346 genes, with 81.89% annotated in public protein databases. The genome data will provide a valuable resource for studying the biology and evolution of Coccinellidae, aiding in pest control strategies and advancing research in the field.

## Background & Summary

The family Coccinellidae, consisting of small beetles, is widely distributed and recognized by various common names, such as ladybugs, ladybirds, or lady beetles. Worldwide, there are over 6,000 described species classified across approximately 360 genera^1^. Coccinellids undergo complete metamorphosis, a holometabolous development that encompasses distinct stages, including the egg, larva, pupa, and adult stages^2^. The adult ladybugs exhibit an oval shape with a rounded back and a flat underside. Notably, there is sexual dimorphism within this family, with adult females being larger than males. Many species within this family possess eye-catching aposematic colors and patterns, such as a red body with black spots, serving as warning signs to potential predators, indicating their unpalatability^3^. Ladybugs are renowned for their beneficial predatory nature, particularly in gardens, agricultural fields, orchards, and similar environments^4^. Both the adult and larval stages of ladybugs actively consume various pest insects^5^. Primarily, ladybirds feed on aphids, while coccids, mites, honeydew, pollen, nectar, and mildew are documented as secondary food sources^6^.

**Fig. 1:**
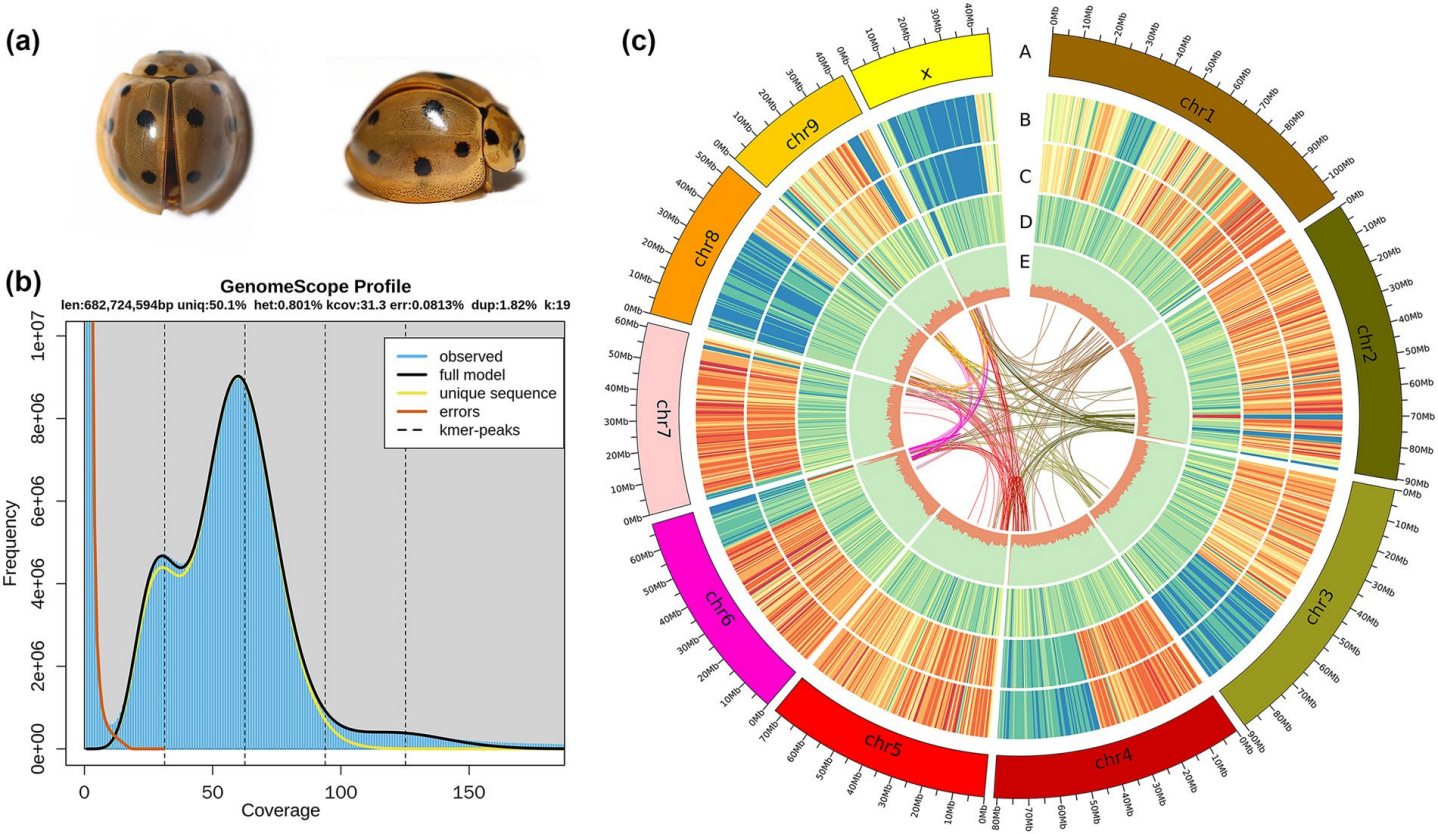
Summary of the final genome assembly results of *Megalocaria dilatata*. (**a**) Photos of *M. dilatata*. (**b**) The K-mer distribution of Illumina paired-end reads using GenomeScope based on a k value of 19. (**c**) A Circos atlas of the chromosomal genome of *M. dilatata*. From the outside to inside rings: (A) Chromosome ideograms (Mb scale); (B) Single-nucleotide polymorphism (SNP) density; (C) Insertion or deletion (INDEL) density; (D) PCG density; (E) GC content. Window size = 500 kb. Syntenic blocks are depicted by connected lines.

*Megalocaria dilatata* (Fabricius, 1775)^7^ belongs to the subfamily Coccinellinae and tribe Coccinellini (Fig. 1a). This species was originally named *Coccinella dilatata*^7^ and later described as *Caria dilatata*^8^, but the widely used name for this species was *Anisolemnia dilatata*^9^. However, the use of the name *Anisolemnia* was subsequently restricted to its type species, and the other species, including *M. dilatata*, were transferred to the genus *Megalocaria*^10^. The giant ladybug *M. dilatata* is known for its large body size and preference for feeding on woolly aphids, which infest bamboo plants and sugarcane^11^. Its distribution is limited to South Asia and the Asia-Pacific regions^11^. The species’ longer lifespan compared to other ladybug species, high reproductive potential, and efficient predation on aphids indicate its significant potential as a biological control agent against pest insects^12^.

Ladybugs have been studied as genetic models since the early 20th century, primarily due to their genetically polymorphic color patterns. The majority of genetic research has focused on color pattern polymorphism in coccinellids^1^. Despite the high species diversity within Coccinellidae, the genomes of only a few species have been sequenced, such as *Coccinella septempunctata*, *Propylea japonica*, *Harmonia axyridis*, *Adalia bipunctata*, *Halyzia sedecimguttata*, *Cryptolaemus montrouzieri*, and *Henosepilachna vigintioctomaculata*^13–20^. Estimations of genome size in Coccinellidae, based on haploid nuclear DNA content or C-value, have revealed greater variation compared to other beetle families^21^. However, there is minimal intraspecific variation in genome size^21^. Genome size in Coccinellidae does not correlate with body size or chromosome number, but larger genomes appear to be associated with longer development periods^1^. Regarding chromosome numbers in Coccinellidae, the diploid count varies across subfamilies and tribes, ranging from 12 to 28 chromosomes^22,23^. The most common diploid number consists of 18 autosomes and a sex chromosome pair, which is considered the ancestral state for Coleoptera^24,25^. Despite these efforts, the lack of genomic data for ladybugs hinders our understanding of their evolution and limits their use as biological control agents.

In this study, we sequenced and assembled a high-quality chromosome-level genome for *M. dilatata*. To achieve this, we employed a comprehensive approach that involved short-read sequencing from Illumina HiSeq, SMRT (single molecule real-time) sequencing from PacBio (Pacific Biosciences), and Hi-C (high-throughput/resolution chromosome conformation capture) scaffolding technology. Our assembly demonstrates remarkable completeness, offering an invaluable genomic asset for future investigations in the field of Coccinellidae, including molecular and evolutionary studies, as well as potential applications in biological control. Additionally, we conducted a comparative analysis incorporating genomes of other related beetle species to identify genes that have undergone rapid evolution within the *M. dilatata* genome.

## Methods

### Sample preparation

The specimens of *M. dilatata* used in this study were cultivated under controlled laboratory conditions at the Sichuan Academy of Agricultural Sciences in Chengdu, China. Genomic DNA for de novo sequencing was extracted from a female adult of *M. dilatata*. The adult specimen was carefully rinsed with 75% ethanol and subsequently dissected on a sterile workbench. Muscle tissue was carefully collected and promptly preserved in liquid nitrogen, with the samples subsequently stored at −80 °C until further analysis. The extraction of high molecular-weight genomic DNA from the muscle tissue was performed using the TIANGEN Blood & Tissue Kit (Tiangen, Beijing, China) in accordance with the manufacturer’s guidelines.

The female adult of *M. dilatata* was used to extract total RNA, which was promptly preserved by freezing it in liquid nitrogen. For library construction, the SMRTbell™ Template Prep Kit 1.0 (Illumina Inc., San Diego, CA, USA) was employed following the recommended protocols provided by the manufacturer. The library was sequenced on an Illumina PacBio platform, generating paired-end reads of 300 base pairs as per the manufacturer’s instructions. The RNA library preparation and sequencing procedures were conducted at Shanghai Personal Biotechnology Co., Ltd. (Shanghai, China). Subsequently, all reads obtained underwent quality control measures, and the Trinity software package^26^ was utilized with default parameters to assemble the transcripts.

### Illumina sequencing and genome size estimation

We prepared a paired-end library with an insert size of 400 bp using the Illumina TruSeq DNA PCR-free prep kit (Illumina Inc., San Diego, CA, USA). To assess the quality of the library, we employed the Agilent 2100 Bioanalyzer (Agilent Technologies, Santa Clara, CA, USA) along with the Agilent High Sensitivity DNA Kit (Agilent Technologies, Santa Clara, CA, USA). Quantification of the library was carried out using the Promega QuantiFluor in conjunction with the Quant-iT PicoGreen dsDNA Assay Kit (Thermo Fisher Scientific, Waltham, MA, USA). Subsequently, the library underwent sequencing on an Illumina NovaSeq 6000 platform (Illumina Inc., San Diego, CA, USA), generating paired-end reads of 150 base pairs in accordance with the manufacturer’s instructions. The DNA library preparation and sequencing procedures were performed at Shanghai Personal Biotechnology Co., Ltd. (Shanghai, China). We initially obtained approximately 62.3 Gb of Illumina DNA raw data^27^ (Table S1).

We performed a quality assessment of the raw data using Fastqc v0.11.8 (http://www.bioinformatics.babraham.ac.uk/projects/fastqc/). To eliminate any undesirable data, we utilized Fastp v0.20.0^28^, employing a base-calling strategy. This process involved the removal of reads with adapter contamination, as well as the exclusion of low-quality reads with a mean PHRED score below 20%. Additionally, any paired reads with a length shorter than 150 bp and reads containing poly-N were also filtered out. To prevent any potential external contamination, a subset of 10,000 high-quality reads was randomly selected. These reads were then subjected to a homologous alignment with publicly available data in GenBank, focusing on identifying closely related species to *M. dilatata*. After quality control, we obtained approximately 60 Gb of high-quality data (Table S2).

For genome size estimation, a total of 59.9 Gb of data obtained from the 400-bp paired-end Illumina library was utilized. The distribution of k-mer copy number (KCN) was calculated to perform this estimation. Specifically, we conducted a 19-mer frequency distribution analysis using Jellyfish v2.3.0^29^, resulting in a count of 682,724,594 19-mers. Based on the unique k-mer depth observed (Fig. 1b), we estimated the genome size of *M. dilatata* to be 0.682 Gb using the GenomeScope package^30^. Furthermore, the sequenced individual exhibited an estimated repetitive fraction of 49.9% and a heterozygosity rate of 0.8% (Table S3).

### PacBio library preparation and sequencing

Whole-genome sequencing was conducted using the PacBio Sequel II platform (Pacific Biosciences, Menlo Park, CA, USA), which utilizes long-read technology. A 15-kb library was prepared following the standard PacBio Template Prep Kit 1.0 preparation protocol (Pacific Biosciences, Menlo Park, CA, USA). For genome sequencing, a SMRT cell was utilized on the PacBio Sequel II sequencing platform, resulting in a total of 34,806,299 long sequencing reads (Table S4), which included 2,767,560 HiFi reads^31^ (Table S5).

### Hi-C library preparation and sequencing

The Hi-C library was prepared using the TruSeq DNA PCR-free prep kit (Illumina Inc., San Diego, CA, USA) following a standard procedure. This process involved various steps, such as DNA crosslinking, restriction enzyme cutting, end filling and biotin marking, ligation, DNA purification and shearing, biotin pull-down, and sequencing using paired ends. Subsequently, the Hi-C library was quantified and sequenced on the Illumina NovaSeq platform, generating paired-end reads of 2 × 150 bp. We obtained approximately 49.6 Gb of sequencing data from the Hi-C library (Table S6). To ensure data quality, a quality check of the Hi-C raw data^32^ was performed using Fastqc v0.11.8, which included assessments of base quality distribution, sequence content, GC distribution, and sequence base quality across all sequences. Furthermore, to generate high-quality Hi-C data, Fastp v0.20.0 was employed with a base-calling strategy, enabling the removal of low-quality reads. Following data filtering with Fastp, we obtained approximately 44.5 Gb of high-quality data (Table S7). In addition, we obtained approximately 89 Gb of transcriptomic data^33^ to assist in genome annotation (Table S8).

### Genome assembly and Hi-C scaffolding

The *M. dilatata* genome was assembled using sequencing reads from Illumina, PacBio, and Hi-C technologies. PacBio sequence data were utilized for the initial de novo assembly, and subsequent polishing was performed using Illumina sequence data to enhance the quality of the contigs. Hi-C reads were employed to scaffold the draft genome. To align the Hi-C data, HiC-PRO v2.5.0^34^ was employed. The junction site of the enzyme was set as ‘GATCGATC’, and default parameters were used for the alignment. The HiC-PRO software conducted two alignment steps. First, the clean paired-end reads were mapped to the draft assembled genome using Bowtie v2.3.2^35^. Second, the unmapped reads with junction fragments were mapped to the draft genome after removing the 3′ end. The results of both mapping steps were merged into a single alignment file. The alignment process of Hi-C data generated approximately 61.8 million uniquely mapped paired-end reads, of which 79% were determined to be valid interaction pairs (Table S9) according to the HiC-Pro pipeline^34^.

**Fig. 2:**
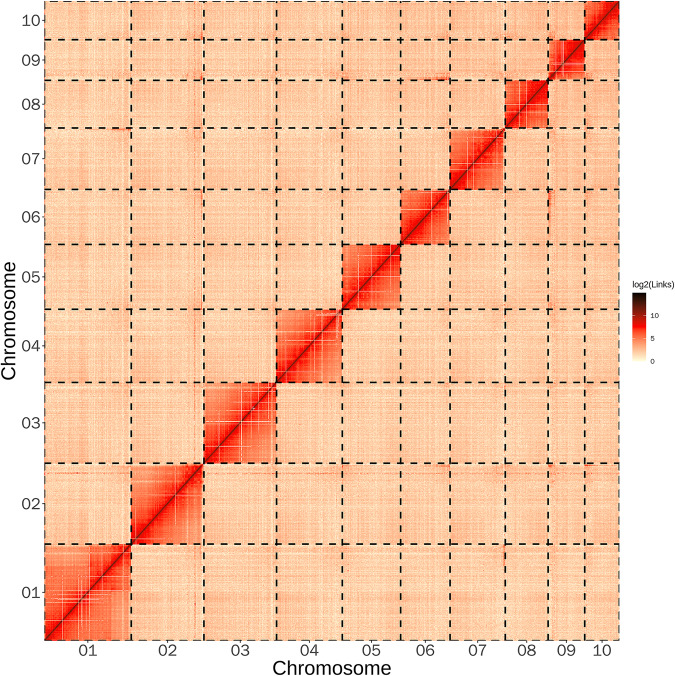
Genome‐wide Hi‐C heatmap for *Megalocaria dilatata* showing interactions among the 10 chromosomes. The number of log2 links was calculated. The color bar indicates the frequency of Hi‐C interaction links from yellow (low) to dark (high) in the plot.

Using the valid Hi-C data, the Endhic de novo assembly pipeline^36^ was employed to generate chromosome-level scaffolds. Endhic was chosen due to its suitability for larger contigs (>1 Mb), providing higher accuracy, fewer parameters, and faster processing compared to other commonly used software. A total of 716,341,908 base pairs were successfully mapped to the chromosomes by Endhic, accounting for 92.21% of the total genome length. To improve the accuracy of the assembly, two rounds of error correction were performed using Illumina reads and the PILON program^37^. As a result, we were able to accurately anchor the Hi-C-assisted genome assembly onto 10 pseudochromosomes (chromosomes), with lengths ranging from 43.35 to 108.16 Mb (Figs. 1c, 2, Table S10). The diploid chromosome number of *M. dilatata* (2n = 20) is consistent with the prevailing chromosome count found in Coccinellidae, which includes 18 autosomes and a pair of sex chromosomes. Notably, this chromosome number is identical to that observed in recently sequenced ladybugs, namely, *C. septempunctata*, *H. sedecimguttata*, and *P. japonica*^13,14,20^. The conservation of this chromosome number across these ladybug species suggests potential evolutionary significance and a shared genetic foundation among members of Coccinellidae.

After applying various refining techniques, such as Pilon polishing, redundancy and contaminant removal, as well as Hi-C scaffolding, we determined the final genome size of *M. dilatata* to be ca. 772.3 Mb, with a scaffold N50 length of ca. 72.48 Mb and a GC content of ca. 34.23% (Table 1). These results closely match the earlier estimations of genome size based on k-mer analysis, validating the accuracy and reliability of our assembly process.

**Table 1:** Statistics of the genome of *Megalocaria dilatata*.

Property	Number
Min sequence length (bp)	14,547
Max sequence length (bp)	106,370,957
Total sequence number	812
N20 (bp)	90,387,075
N20 number	2
N50 (bp)	72,475,326
N50 number	5
N90 (bp)	43,284,452
N90 number	10
N number	26,807
N rate %	3.47106588454537e-05
Total sequence length (bp)	772,298,795
GC content %	34.2261446879507
Sequences greater than 1 kb	812

### Repetitive element analysis

Repetitive elements (REs) were detected through two approaches: homology search and de novo annotation. The homology-based search was conducted against RepBase-20150807, utilizing RepeatMasker v4.0.5^38^. For de novo repeat library construction, RepeatModeler v1.0.4^38^ was employed. Protein-coding-like sequences that exhibited similarity to those in the Swiss-Prot database were eliminated from the de novo library. Finally, RepeatMasker v4.0.5^38^ was used to search the constructed de novo library. We identified a total of 493.33 Mb of repeat sequences (Table S11), which accounted for 63.88% of the genome. The *M. dilatata* genome had a much higher level of repetitive sequence than the genomes of other sequenced coleopterans^14^, which raises intriguing questions about the evolutionary forces shaping genome architecture in this species. Among these repeats, DNA elements were the most prevalent, constituting 175.13 Mb (22.68%). This finding aligns with previous studies on *Aedes albopictus* (Skuse, 1894)^39,40^ and *P. japonica*^14^, which also reported DNA elements as the predominant repetitive elements. Additionally, 113.97 Mb (14.76%) were attributed to long interspersed nuclear elements (LINEs), 9.54 Mb (1.23%) to long terminal repeat elements (LTRs), and 3.11 Mb (0.4%) to short interspersed nuclear elements (SINEs). The DNA elements and LINEs may contribute to genomic plasticity and adaptation. Their abundance might be linked to specific biological features, such as responses to environmental changes, host-pathogen interactions, or other ecological factors. Moreover, we observed 191.59 Mb (24.81%) of unclassified repeat elements that could not be categorized into known repeat elements. The remaining repeats included 1 Kb of small RNA, 312.05 Kb of satellites (0.04%), 375.87 Kb of simple repeats, and 2 Kb of low complexity repeats. These repeats could be utilized in population genetics, phylogenetics, or association studies, providing a bridge between the observed genomic composition and phenotypic traits. Notably, DNA elements and LINEs were the two most abundant repeat categories in the *M. dilatata* genome, collectively accounting for 37.44% of the genome assembly.

### Protein-coding gene prediction

The prediction of protein-coding genes (PCGs) within the genome involves multiple approaches, including *ab initio* prediction, homology-based prediction, and evidence from transcriptome-based prediction. *Ab initio* gene prediction was carried out using Augustus v3.03^41^, GeneID v1.4^42^, and GeneMark v4.35^43^. In the homology-based approach, amino acid sequences from related ladybugs were aligned to the *M. dilatata* assembly using Exonerate v2.2.0^44^. Transcriptome-based prediction methods utilized RNA-seq data. The assembled transcripts from Trinity were used as inputs for gene model prediction using PASA^45^. To integrate all the gene prediction results, EVidenceModeler (EVM) vr2012-06-25^46^ was employed to generate consensus gene models. We predicted a total of 25,346 genes in *M. dilatata*, with an average gene length of 16,245 bp (Table S12). These genes comprised 101,803 exons, with an average of 4 exons per gene. The genome contained 101,803 coding sequences (CDSs), with an average CDS length of 305.1 bp. The total length of CDSs reached 31,069,524 bp, representing 4.0229% of the genome.

### Noncoding RNA prediction

The identification of transfer RNA (tRNA) genes within the genome was performed using tRNAscan-SE v1.3.1^47^. For the prediction of ribosomal RNA (rRNA) genes, RNAmmer v1.2^48^ was utilized. To identify the remaining noncoding RNA (ncRNA) genes, a search was conducted against the Rfam database using the Infernal v1.1.3 program^49^. We identified 15,915 ncRNAs in the genome, including 1,327 8 S rRNAs, 1,418 18 S rRNAs, 1,314 28 S rRNAs, 9,328 tRNAs, and 2,528 other ncRNAs (Table S13).

### Gene function annotation

Gene functions were assigned based on the most significant alignments against the Swiss-Prot and NCBI nonredundant protein sequence (NR) databases using local Blastp with a threshold E-value of 1e-5. Additionally, the Pfam database was searched through InterProScan^50^ for gene function annotation. InterProScan^50^ was also employed to obtain domain information and perform GO (Gene Ontology) term annotation. The predicted protein sequences were submitted to KAAS (KEGG Automatic Annotation Server) to obtain KEGG (Kyoto Encyclopedia of Genes and Genomes) pathway annotations^51^. For the prediction of carbohydrate-active enzymes (CAZy), Hmmer v3.0^52^ was utilized. An E-value cut-off of 1e-5 was used for sequences longer than 80 amino acids, and an E-value cut-off of 1e-3 was used for sequences shorter than 80 amino acids.

Among the 25,346 predicted genes, 20,397 (80.47%) exhibited hits to proteins in the Nr database, 11,148 (43.98%) in the Swiss-Prot database, and 14,751 (58.20%) in the Pfam database (Table S14). Additionally, 10,663 (42.07%) genes were annotated with GO terms (Fig. S1), while 6,505 (25.66%) genes were annotated with KEGG Orthology (KO) terms (Fig. S2). Overall, a total of 20,756 (81.89%) genes were annotated by at least one of the public databases, indicating substantial functional annotation coverage.

Furthermore, CAZy annotation predicted the presence of various carbohydrate-active enzymes in the genome (Fig. S3). Specifically, we identified 150 genes of glycosyl transferases (GT), 3 genes of polysaccharide lyases (PL), 71 genes of carbohydrate esterases (CE), 40 genes of auxiliary activities (AA), 59 genes of carbohydrate-binding modules (CBM), and 136 genes of glycoside hydrolases (GH). The identification of carbohydrate-active enzymes through CAZy annotation suggests their involvement in various metabolic processes related to carbohydrate metabolism, potentially reflecting the dietary preferences and ecological niche of *M. dilatata*.

## Data Records

The genomic (Illumina, PacBio, Hi-C) and transcriptomic sequencing data was deposited at the NCBI Sequence Read Archive (SRA) database under BioProject ID PRJNA1029341^53^. The accession numbers of the Illumina sequencing data, HiFi sequencing data, Hi-C sequencing data, and transcriptomic data are SRR26425793^27^, SRR26425792^31^, SRR26425791^32^, and SRR26425790^33^, respectively. The accession number of the genome assembly is JAWQEH000000000^54^. The genome assembly and raw sequencing data are also available at the China National GeneBank DataBase (CNGBdb) under Project ID CNP0004482^55^.

## Technical Validation

To assess the completeness and continuity of the *M. dilatata* genome assembly, we employed Benchmarking Universal Single-Copy Orthologues (BUSCO) v.3.0.2^56^, utilizing the Insecta database comprising 75 species and 1,367 universal single-copy orthologous genes, which evaluates the presence of conserved insect genes. This analysis allows us to assess the comprehensiveness and representation of the insect gene set within the *M. dilatata* genome. By comparing our assembly to a set of 1,367 conserved genes, we identified a remarkable 1,330 genes (97.29%) present in the *M. dilatata* genome (Table S15). This high BUSCO score signifies the robustness and completeness of our assembly, suggesting that a significant majority of the conserved insect genes are accurately represented within the *M. dilatata* genome.

BUSCO v.3.0.2^56^ was also used to assess the completeness of the annotated protein-coding genes, which revealed that 98.76% of the conserved orthologous genes were complete in the predicted PCGs, with 96.27% single-copy and 2.49% duplicated (Table S16). This indicates a comprehensive prediction and annotation of the gene set. The comprehensive results obtained from BUSCO assessment and functional annotation collectively demonstrate that the genes in the newly assembled *M. dilatata* genome are well annotated. This robust annotation provides a solid foundation for further investigations into the functional genomics and comparative studies of this giant ladybug.

### Supplementary information

Supplemental Information for Chromosome-level genome assembly of the giant ladybug Megalocaria dilatata

## Data Availability

The bioinformatic analyses were performed using the manuals and protocols by the software developers. If manually adjusted parameters were used, the software version and method used are described in the Methods.
