# Supplementary material for: Chromosome-level genome assembly of the giant ladybug Megalocaria dilatata
Source: Sci Data. 2024 Jan 24;11:117. doi: 10.1038/s41597-024-02990-1 (PMC10808094; doi:10.1038/s41597-024-02990-1)
Supplement: Supplementary file 1 — Supplemental Information for Chromosome-level genome assembly of the giant ladybug Megalocaria dilatata [file 41597_2024_2990_MOESM1_ESM.pdf]

Table of Contents:

**Table S1**	Page 2
**Table S2**	Page 3
**Table S3**	Page 4
**Table S4**	Page 5
**Table S5**	Page 6
**Table S6**	Page 7
**Table S7**	Page 8
**Table S8**	Page 9
**Table S9**	Page 10
**Table S10**	Page 11
**Table S11**	Page 12
**Table S12**	Page 13
**Table S13**	Page 14
**Table S14**	Page 15
**Table S15**	Page 16
**Table S16**	Page 17
**Figure S1**	Page 18
**Figure S2**	Page 19
**Figure S3**	Page 20

Table S1 Raw data from the Illumina NovaSeq platform.

Reads number	Total bases (bp)	N rate %	GC content %	Q20 rate %	Q30 rate %
412,832,610	62,337,724,110	0.00	34.65	97.68	93.71

Table S2 High-quality data from Illumina sequencing.

HQ reads	HQ reads %	HQ data (bp)	HQ data %
404,350,386	97.95	59,983,784,665	96.22

Table S3 Genome estimation based on 19-mer analysis.

Property	Value
K-mer	19
K-mer depth	62.6
Heterozygosity (%)	0.8%
Genome size (Mb)	682
Repetitive fraction (%)	49.9%
Used bases (G)	59.9
X	88

Table S4 Raw data from the PacBio Sequel platform.

Property	Value
Min sequence length (bp)	100
Max sequence length (bp)	637,684,484
Total sequence number	34,806,299
N20 (bp)	19,246
N20 number	3,241,882
N50 (bp)	14136
N50 number	11,870,138
N90 (bp)	10,091
N90 number	27,454,854
N number	0
N rate %	0.0
Total sequence length (bp)	465,584,870,040
GC content %	35.710860412134025
Sequences greater than 1kb	34,252,578

Table S5 Statistics of HiFi data.

Property	Value
Min sequence length (bp)	124
Max sequence length (bp)	46,542
Total sequence number	2,767,560
N20 (bp)	18,690
N20 number	368,407
N50 (bp)	14,570
N50 number	1,095,782
N90 (bp)	10,566
N90 number	2,367,827
N number	0
N rate %	0.0
Total sequence length (bp)	39,622,057,686
GC content %	34.52743865908267
Sequences greater than 1kb	2,767,469

Table S6 Raw data of Hi-C sequencing.

Reads number	Total bases (bp)	N (%)	GC (%)	Q20 (%)	Q30 (%)
330,575,020	49,586,253,000	0.0308	35.14	95.3	88.48

Table S7 High-quality Hi-C sequencing data.

HQ reads (num.)	HQ reads %	HQ data (bp)	HQ Data %
299,648,808	90.64	44,482,809,713	89.71

Table S8 Statistics of transcriptomic data.

Total sequence number	Total bases (bp)	GC %	Max length (bp)	Min length (bp)	N50 (bp)	Sequence greater than 1 kb
40,435,056	88,985,337,269	39.41	256,506	51	2,347	37,513,112

Table S9 Statistics of valid paired-end reads.

Iterm	Number
Valid interaction pairs	48,851,191
Dangling end pairs	4,108,562
Religation pairs	573,425
Self cycle pairs	220,131
Dumped pairs	8,044,697

Table S10 Chromosome lengths based on Hi-C scaffolding.

Chromosome number	Length (bp)
Chr_01	108,162,212
Chr_02	90,529,952
Chr_03	90,486,835
Chr_04	81,933,781
Chr_05	72,619,149
Chr_06	61,608,430
Chr_07	68,823,457
Chr_08	53,338,595
Chr_09	45,539,394
Chr_10	43,347,103

Table S11 Statistics of repeat elements.

Elements	Number of elements	Length (bp)	Percentage of Genome
SINEs	14,590	3,105,742	0.40%
ALUs	0	0	0.00%
MIRs	7,926	1,622,868	0.21%
LINEs	266,868	113,969,124	14.76%
LINE1	163	26,830	0.00%
LINE2	21,516	15,510,604	2.01%
L3/CR1	7,647	3,834,205	0.50%
LTR elements	12,525	9,535,463	1.23%
ERVL	603	1,114,358	0.14%
ERVL-MaLRs	0	0	0.00%
ERV_classI	184	14,074	0.00%
ERV_classII	6	2,087	0.00%
DNA elements	429,102	175,128,342	22.68%
hAT-Charlie	966	486,734	0.06%
TcMar-Tigger	322	115,082	0.01%
Unclassified	615,026	191,592,058	24.81%
Total interspersed repeats	-	493,330,729	63.88%
Small RNA	4	1,144	0.00%
Satellites	1,701	312,051	0.04%
Simple repeats	496	375,867	0.05%
Low complexity	11	1,995	0.00%

Table S12 Statistics of gene prediction results.

Property	Value
Total genes length	411,746,031
Genes percentage of genome	53.3143%
Total genes number	25,346
Average gene length	16,245
Total exons number	101,803
Average exons per gene	4
Total exons length	31,069,524
Exons percentage of genome	4.0229%
Total CDS number	101,803
Average CDS length	305.1
Average exons length	305.1
Average introns length	4,978.9
Total CDSs length	31,069,524
CDSs percentage of genome	4.0229%
Average transcription length	1,225.8

Table S13 Statistics of noncoding RNAs.

RNA type	Copy	Avg. length (bp)	Total length (bp)	percentage of genome
8s rRNA	1,327	115.06	152,683	0.019769938913345
18s rRNA	1,418	1,866.96	2,647,355	0.342788959032365
28s rRNA	1,314	4,646.42	6,105,390	0.790547653256406
tRNA	9,328	73.63	686,833	0.088933584313051
other ncRNA	2,528	461.06	1,165,551	0.150919696825372

Table S14 Statistics of functional annotation based on various databases.

Annotation in database	Number of genes	Percentage %
GO	10,663	42.07
KO	6,505	25.66
Swiss-Prot	11,148	43.98
Pfam	14,751	58.2
NR	20,397	80.47
At least one database	20,756	81.89

Table S15 BUSCO assessment of the genomic completeness and continuity.

Groups	Numbers	Completeness
Complete BUSCOs (C)	1,330	97.29%
Complete and single-copy BUSCOs (S)	1,303	95.32%
Complete and duplicated BUSCOs (D)	27	1.98%
Fragmented BUSCOs (F)	8	0.59%
Missing BUSCOs (M)	29	2.12%
Total BUSCO groups searched	1,367	100%

Table S16 BUSCO assessment of the gene completeness.

Groups	Numbers	Completeness
Complete BUSCOs (C)	1,350	98.76%
Complete and single-copy BUSCOs (S)	1,316	96.27%
Complete and duplicated BUSCOs (D)	34	2.49%
Fragmented BUSCOs (F)	1	0.07%
Missing BUSCOs (M)	16	1.17%
Total BUSCO groups searched	1,367	100%

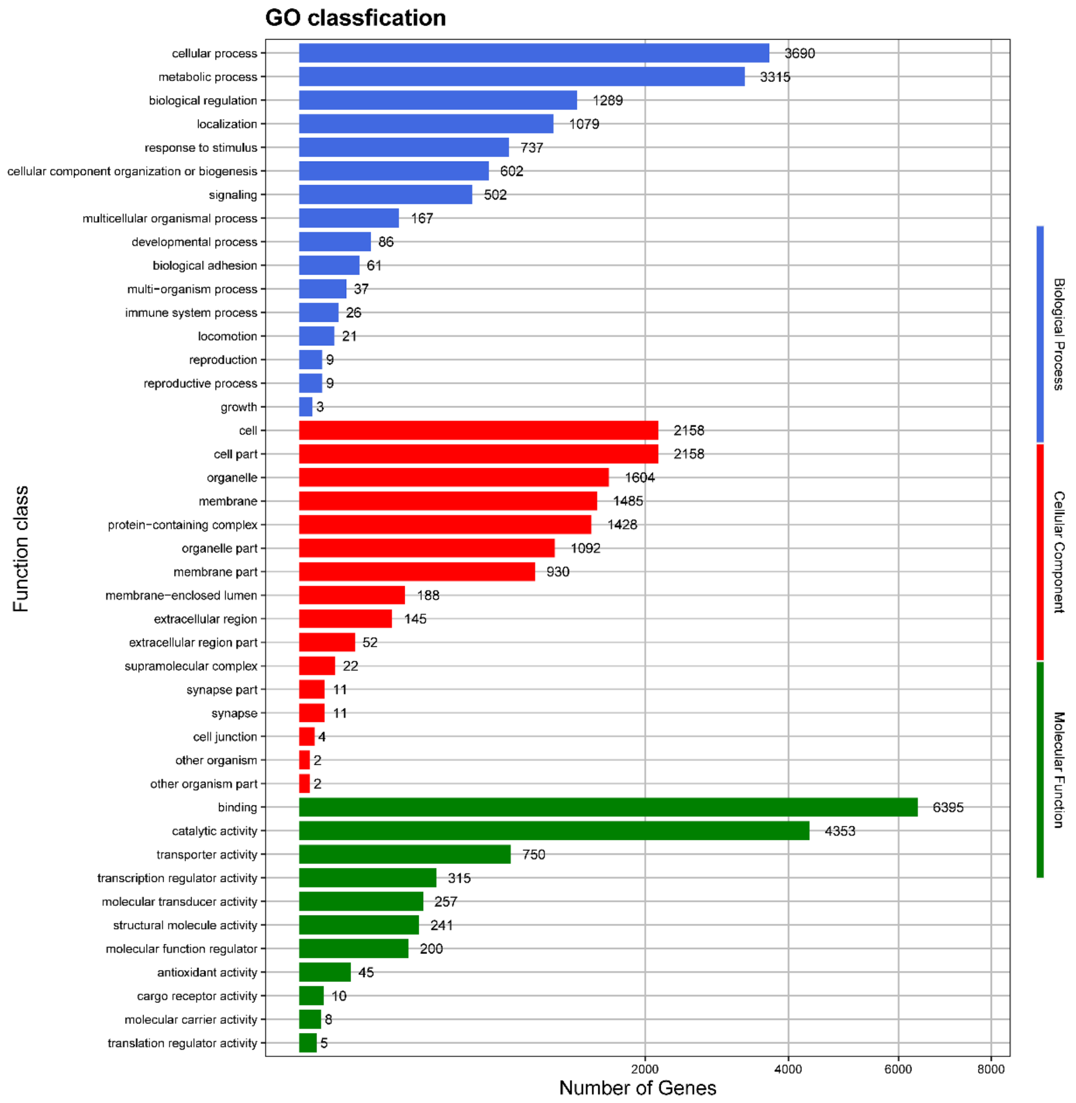

Figure S1. Go classification of PCGs in *Megalocaria dilatata*. For each category, gene number is represented by bar length.

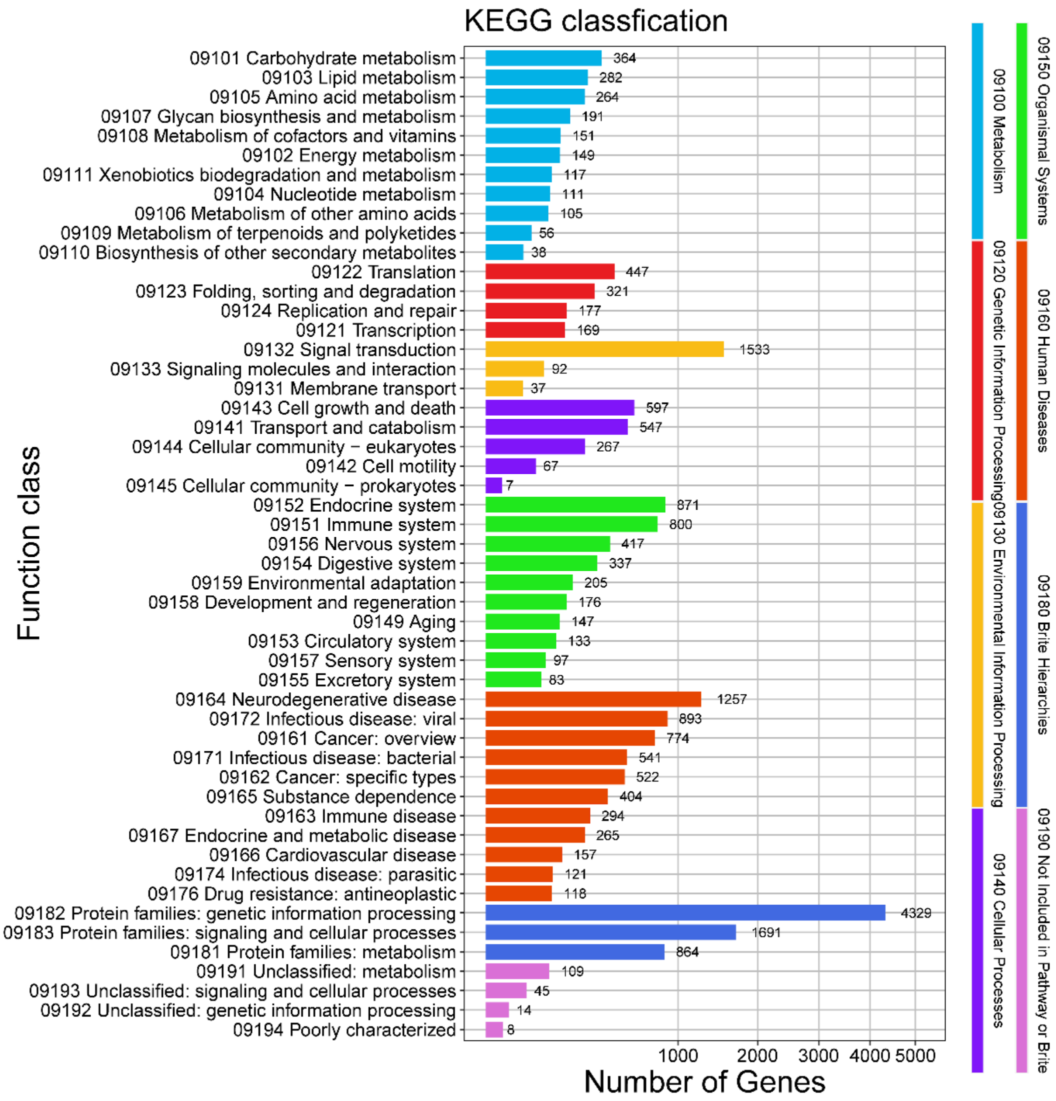

Figure S2. KEGG classification of PCGs in *Megalocaria dilatata*. For each category, gene number is represented by bar length.

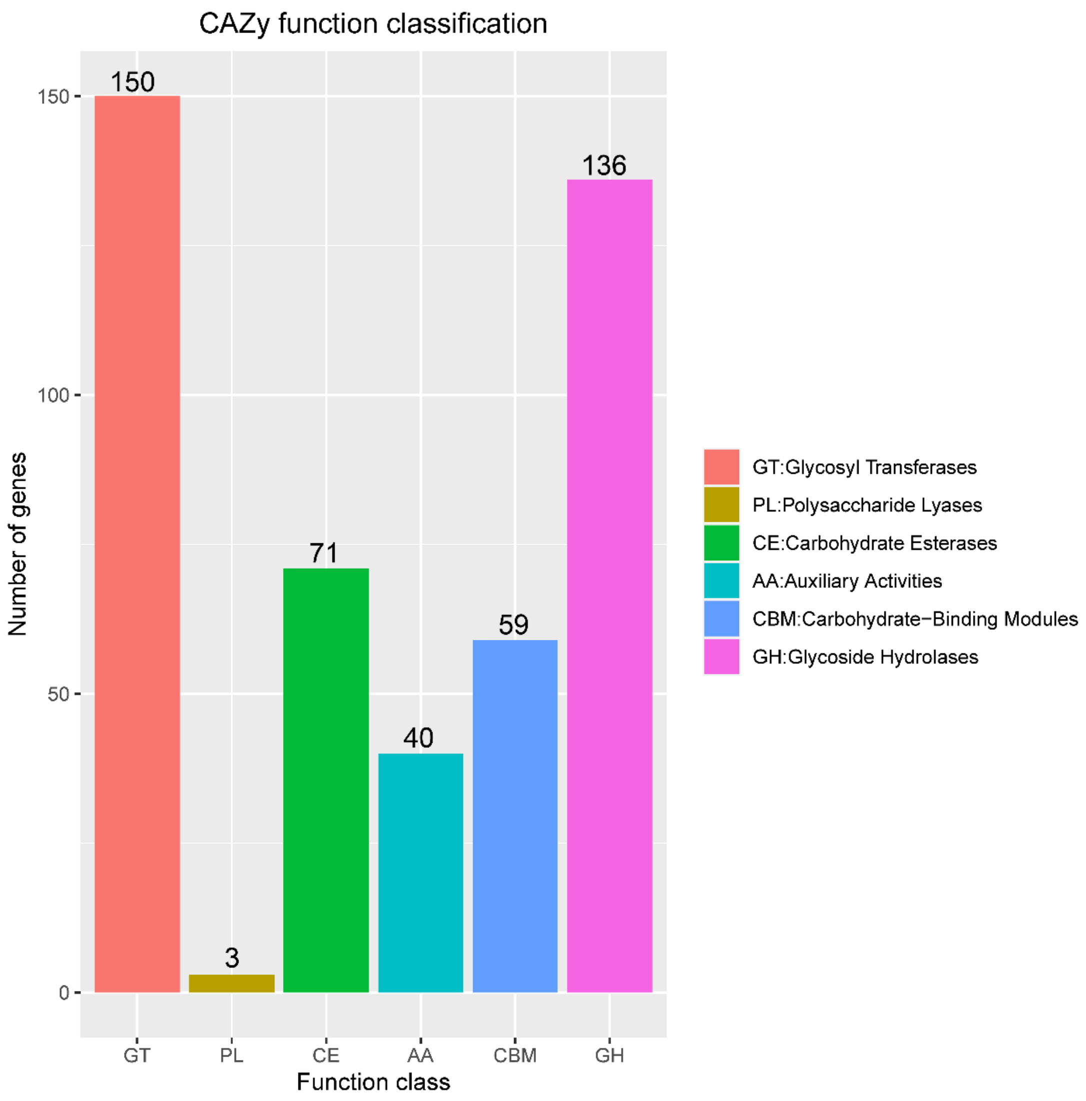

Figure S3. CAZy function classification of genes in *Megalocaria dilatata*. For each category, gene number is represented by bar length.
